# Machine learning model based on the radiomics features of CE-CBBCT shows promising predictive ability for *HER2*-positive BC

**DOI:** 10.1097/MD.0000000000044300

**Published:** 2025-09-12

**Authors:** Xianfei Chen, Minghao Li, Xueli Liang, Danke Su

**Affiliations:** a Department of Medical Imaging Center, Guangxi Medical University Cancer Hospital, Nanning, Guangxi, China; b Department of Radiology, The First Affiliated Hospital of Hainan Medical University, Hainan Medical University, Haikou, China.

**Keywords:** breast cancer, contrast-enhanced cone-beam breast computed tomography, human epidermal growth factor receptor 2, machine learning, radiomics

## Abstract

This study aimed to investigate whether establishing a machine learning (ML) model based on contrast-enhanced cone-beam breast computed tomography (CE-CBBCT) radiomic features could predict human epidermal growth factor receptor 2-positive breast cancer (BC). Eighty-eight patients diagnosed with invasive BC who underwent preoperative CE-CBBCT were retrospectively enrolled. Patients were randomly assigned to the training and testing cohorts at a ratio of approximately 7:3. A total of 1046 quantitative radiomics features were extracted from the CE-CBBCT images using PyRadiomics. *Z*-score normalization was used to standardize the radiomics features, and Pearson correlation coefficient and one-way analysis of variance were used to explore the significant features. Six ML algorithms (support vector machine, random forest [RF], logistic regression, adaboost, linear discriminant analysis, and decision tree) were used to construct optimal predictive models. Receiver operating characteristic curves were constructed and the area under the curve (AUC) was calculated. Four top-performing radiomic models were selected to develop the 6 predictive features. The AUC values for support vector machine, linear discriminant analysis, RF, logistic regression, adaboost, and decision tree were 0.741, 0.753, 1.000, 0.752, 1.000, and 1.000, respectively, in the training cohort, and 0.700, 0.671, 0.806, 0.665, 0.706, and 0.712, respectively, in the testing cohort. Notably, the RF model exhibited the highest predictive ability with an AUC of 0.806 in the testing cohort. For the RF model, the DeLong test showed statistically significant differences in the AUC between the training and testing cohorts (Z = 2.105, *P* = .035). The ML model based on CE-CBBCT radiomics features showed promising predictive ability for human epidermal growth factor receptor 2-positive BC, with the RF model demonstrating the best diagnostic performance.

## 1. Introduction

Breast cancer (BC) is a heterogeneous disease that is characterized by diverse molecular subtypes and biological characteristics. Each molecular subtype has a unique treatment method, response, and survival outcome.^[1]^ Approximately 15% to 20% of BCs are human epidermal growth factor receptor 2 (*HER2*)-positive. This subtype is known for its aggressive nature, poor prognosis, short survival time, and high recurrence rate.^[2]^ However, the clinical use of monoclonal antibody drugs targeting *HER2*, such as trastuzumab, has remarkably improved the diagnosis, treatment, and prognosis of *HER2*^+^ BC.^[3,4]^ Accurate assessment of *HER2* positivity is crucial in selecting individuals who can benefit from *HER*2-targeted therapy and improving outcomes for patients with *HER2*^+^ BC. This is especially important in cases that are initially challenging to resect and that require neoadjuvant chemotherapy.

The gold standard assessment of *HER2*^+^ BC relies on immunohistochemistry (IHC) or fluorescence in situ hybridization (FISH).^[5]^ These methods often require a core needle biopsy or invasive surgery. However, needle biopsy samples may not fully represent the entire tumor because of the relatively small number of tissue samples obtained and the tumor heterogeneity.^[6]^ Moreover, genetic heterogeneity within individual tumors significantly contributes to drug resistance and treatment failure.^[7]^ Additionally, the discrepancy rate between limited biopsy and surgical specimens ranges from 3.5% to 14%.^[8]^ Therefore, it is crucial to develop an accurate and noninvasive method to assess HER2 positivity in patients with BC.

Contrast-enhanced cone-beam breast computed tomography (CE-CBBCT) is a new imaging modality that combines the advantages of mammography and magnetic resonance imaging (MRI). It simultaneously provides information on calcifications, morphology, and quantitative blood flow characteristics of lesions,^[9]^ with diagnostic efficacy comparable to MRI.^[10]^ Radiomics transforms digital medical images into easily accessible multidimensional quantitative imaging features that can reveal intratumoral heterogeneities. Therefore, it has gained attention among patients with cancer owing to its potential in precision medicine.^[11,12]^ Machine learning (ML)^[13,14]^ is a branch of artificial intelligence that utilizes advanced algorithms to identify and analyze subtle features of lesions, which may be challenging for the human eye. Previous studies have demonstrated the potential of combining radiomic features with ML models to predict *HER*2 positivity in BC patients. However, these studies mainly focused on the radiomic features obtained from mammography,^[15]^ ultrasound,^[16]^ and MR,^[17]^ with area under the curve (AUC) values ranging from 0.778 to 0.8054. No published studies have reported the accuracy of ML models combined with CE-CBBCT-based radiomics for predicting *HER*2 status in patients with BC. We hypothesized that the fusion of radiomic signatures and ML could accurately assess *HER*2 status in patients with invasive breast cancer (IBC).

Thus, the primary objective of this study was to develop and validate ML models based on CE-CBBCT radiomics to predict HER2^+^ IBC. Furthermore, we compared the performance of various machine learning methods to identify the optimal predictive model.

## 2. Materials and methods

### 2.1. Participants

This retrospective study was approved by the *Guangxi Medical University Cancer Hospital* Institutional Review Board, which waived the requirement for informed consent (ethics approval number: LW2023073). CE-CBBCT images and clinicopathological data of patients with IBC referred to the Guangxi Medical University Cancer Hospital (Nanning, China) between January 2021 and January 2022 were reviewed.

**Figure 1.**
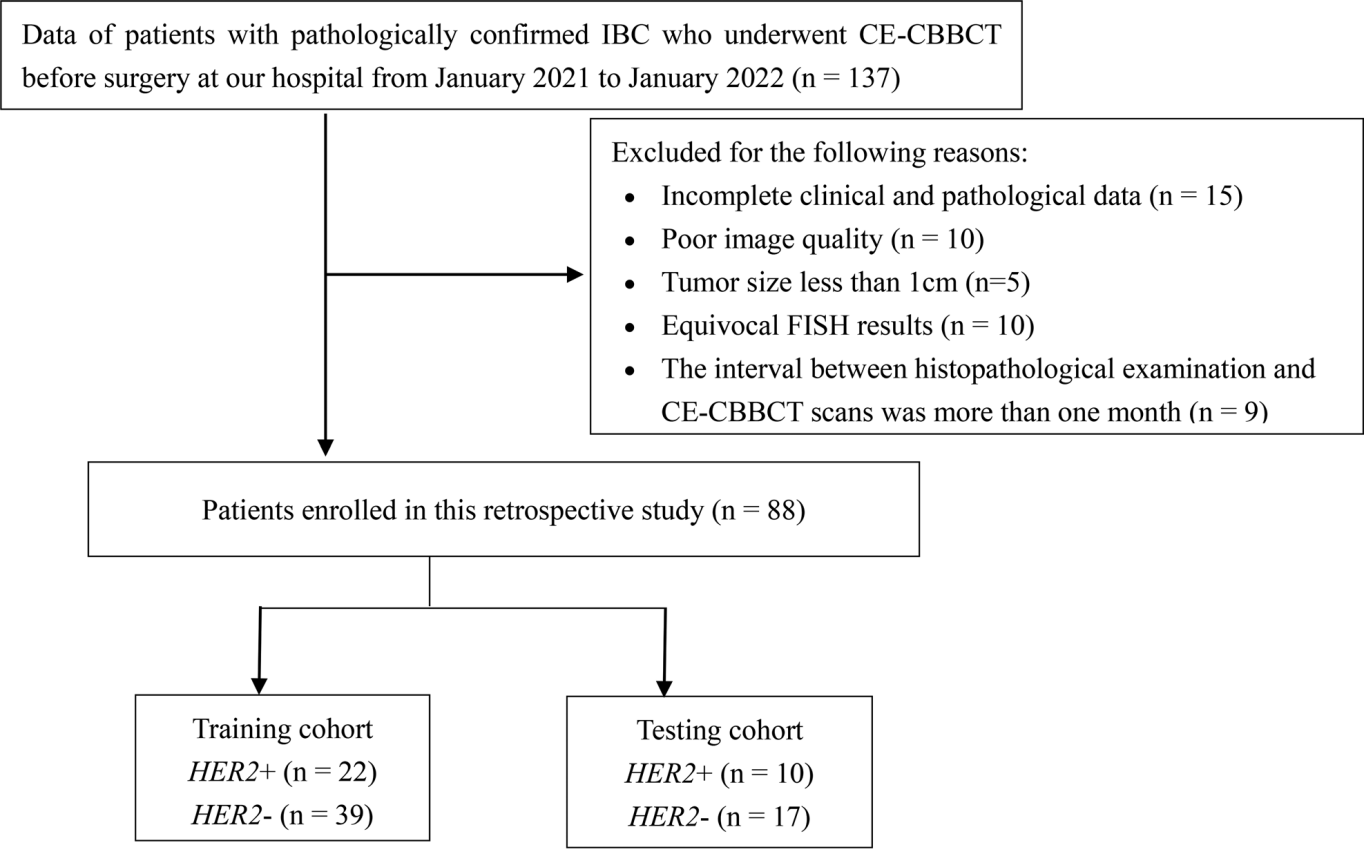
Flow diagram of patient recruitment.

The inclusion criteria for this study were: CE-CBBCT examination conducted 2 weeks before biopsy or resection.^[18]^ No surgery or treatment was performed before CE-CBBCT. The pathological diagnosis of all patients was based on surgical resection or needle biopsy. Patients with pathologically confirmed IBC. Conversely, the exclusion criteria were as follows: tumor size of <1 cm. Complete clinical and pathological data. Poor image quality. The interval between histopathological examination and CE-CBBCT scans was >1 month.^[19]^ Equivocal FISH results. Figure 1 shows the flow diagram of this study.

### 2.2. Histopathology and immunohistochemistry study

*HER2* positivity was evaluated using IHC or FISH according to the guidelines established by the American Society of Clinical Oncology/College of American Pathologists.^[20]^ If the IHC result was 0 or 1+, the patient was considered *HER2*^−^; if the result was 3+, the patient was considered *HER2*^+^. If the IHC results were 2+, additional FISH analysis was necessary to determine gene amplification. If the results were positive, the patient was defined as *HER2*^+^. For the estrogen receptor/progesterone receptor examination, tumors were categorized as estrogen receptor/progesterone receptor^+^ if nuclear staining was present in ≥1% of tumor cells. A critical threshold of 20% was set for Ki-67 expression, with tumors ≥20% considered highly expressed.

### 2.3. CBBCT protocols

All cone-beam breast computed tomography (CBBCT) examinations in this study were conducted using a dedicated flat-panel breast CT scanner (KBCT 1000, Tianjin Kening Medical Devices Co., Ltd, Tianjin, China) that was approved by the Food and Drug Administration of both the U.S. and China. The patients lay in a prone position with the breast hanging naturally in an open scanning area. A constant voltage of 49 kVp and alternating current of 50 to 160 mA were set according to breast size and density.^[21,22]^ Non-contrast-enhanced CBBCT was then performed. An intravenous bolus of 80-mL nonionic iodine contrast agent was injected at a flow rate of 2.0 mL/s using a dual-chamber power injector (MEDRAD Stellant D-CE, BAYER, Bayer Medical Care Inc., Indianola). Two separate post-contrast CBBCT scans were conducted 60 and 120 seconds after the start of the contrast agent injection.^[19,23,24]^

### 2.4. Image study

Two radiologists with 15 and 5 years’ experience in breast imaging, including CBBCT, jointly assessed the imaging characteristics of the patients. Both doctors were blinded to the patients’ pathological findings. CBBCT characteristics were evaluated and recorded according to the MRI and mammography sections of the American College of Radiology Breast Imaging Reporting and Data System atlas and lexicon.

### 2.5. Tumor segmentation

**Figure 2.**
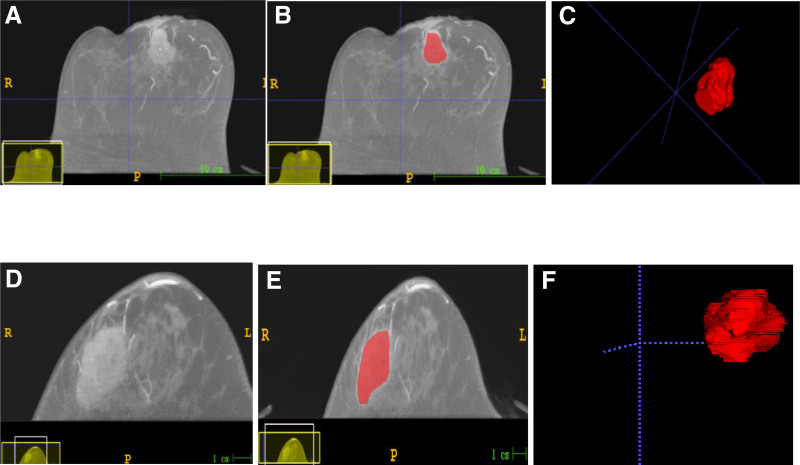
Three-dimensional manual segmentation of the tumor. (A)–(C) A 61-year-old female confirmed to have *HER2*^+^ IBC. (D)–(F) A 49-year-old female confirmed to have *HER2*^−^ IBC. HER2 = human epidermal growth factor receptor 2, IBC = invasive breast cancer.

According to previous studies,^[19,23,24]^ the contrast between the BC tumor and the background peaked 60 to 120 seconds after contrast administration. This study chose region of interest (ROI) segmentation during the first phase of CE-CBBCT. To perform three-dimensional (3D) ROI segmentation, ITK-SNAP software (version 3.6; http://www.itksnap.org) was used. The regions of necrosis were excluded during segmentation. Figure 2 provides a detailed description of the process. Doctor 1, a radiologist with 15 years of experience, manually delineated the 3D-ROI. To minimize the influence of the partial-volume effect, the boundaries of the selected ROIs were slightly smaller than those observed with the naked eye.^[19]^ CBBCT images from 30 patients were randomly selected 1 month later to test the reproducibility and stability of the CBBCT imaging characteristics. Doctor 1 and another radiologist (Doctor 2) with years of experience repeated the segmentation. Intra-observer and interobserver correlation coefficients (ICCs) were calculated to assess the consistency and reproducibility of characteristics. ICC > 0.75 indicated good consistency.^[18,25]^

### 2.6. Radiomics feature extraction

Several image preprocessing steps were performed to enhance the abundance of features before extracting radiomics features. These steps include gray-level discretization, gray-level normalization (GLN), and voxel resampling.^[18,25,26]^ More details are described in the File S1, Supplemental Digital Content, https://links.lww.com/MD/P951.

Wavelet imaging filters and Laplacian of Gaussian (LoG) filtering were used to process the original images and generate additional images, and 1046 quantitative radiomics features were extracted using PyRadiomics (https://github.com/Radiomics/pyradiomics).^[27]^ including 18 first-order statistical features, 14 shape-based features (3D), 68 texture features, 688 wavelet features, and 258 LoG features. Radiomic features were extracted and standardized following the Imaging Biomarker Standardization Initiative.^[28]^ Detailed information on the formulae for calculating the radiomics signatures can be found at https://pyradiomics.readthedocs.io.

### 2.7. Feature selection and model evaluation

The patients were randomly assigned to the training and testing cohorts at a ratio of approximately 7:3. The Synthetic Minority Oversampling Technique^[29]^ resampling method is used to address class imbalances. *Z*-score normalization was used to standardize radiomic features. To reduce the dimensionality of all radiomic features, Pearson correlation coefficient^[30]^ was used to identify the highly correlated feature pairs. If the absolute Pearson correlation coefficient was ≥0.99, then one feature was removed. Subsequently, a one-way analysis of variance^[25]^ was performed to identify significant features, 20 of which were selected. Six supervised classification algorithms (linear discriminant analysis, random forest [RF], support vector machine [SVM], logistic regression [LR], AdaBoost [AB], and decision tree [DT]) were used to construct the prediction models. A 10-fold cross-validation method was used to assess the accuracy of the models.

To evaluate the model performance, receiver operating characteristic (ROC) curves were drawn, and the AUC, accuracy, sensitivity, specificity, positive predictive value, and negative predictive value were calculated using FeAture Explorer Pro (FAE; v0.5.6) in Python v3.7.6. The FAE code can be found at https://github.com/salan668/FAE.^[31]^

### 2.8. Statistical analysis

Shapiro–Wilk and *F* tests assessed the normality and homogeneity of variance for continuous variables. Continuous variables exhibiting normal distributions are presented as mean ± standard deviation and were subjected to independent-sample *t* tests for comparison. Conversely, non-normally distributed variables are presented as medians (lower quartile, upper quartile) and compared using the Mann–Whitney *U* test. Classification data are expressed in terms of relative distribution frequency and percentage. Categorical variables were compared using either the *χ*^2^ test or Fisher exact test. ROC curves were compared using the DeLong method. All statistical analyses were performed using SPSS v26.0 (IBM Corp., Armonk). *P* < .05 indicated that the differences were considered statistically significant at *P* < .05.

## 3. Results

### 3.1. Clinicopathological and CE-CBBCT characteristics

Ultimately, 88 consecutive patients were enrolled in the study. In the training cohort, 22 (36.1%) patients were *HER2*^+^, and 39 (63.9%) were *HER2*^−^; in the testing cohort, 10 (37.0%) were *HER2*^+^ and 17 (62.9%) were *HER2*^−^. Axillary lymph node involvement differed significantly between the testing and training cohorts (*P* = .042), whereas no significant differences were found in any other clinicopathological characteristics between the 2 cohorts (Table 1). No significant differences in the CBBCT findings were observed between the testing and training cohorts (Table 2).

**Table 1:** Clinicopathological characteristics of patients in the training and testing cohorts.

Characteristic	Training cohort (n = 61)	Testing cohort (n = 27)	*t*/*χ*^2^ value	*P* value
Age (yr)	49.31 ± 9.34	53.00 ± 12.49	−1.536	.128
Diameter (cm)	2.90 (2.20–4.10)	2.70 (2.30–4.00)	−0.412	.680
Menstrual status, n (%)			0.392	.531
Premenopausal	36 (59.0%)	14 (51.9%)		
Postmenopausal	25 (41.0%)	13 (48.1%)		
ER, n (%)			0.713	.399
Positive	48 (78.7%)	19 (70.4%)		
Negative	13 (21.3%)	8 (29.6%)		
PR, n (%)			1.045	.307
Positive	47 (77.0%)	18 (66.7%)		
Negative	14 (23.0%)	9 (33.3%)		
Ki-67, n (%)			0.017	.897
≤20%	9 (14.8%)	5 (18.5%)		
>20%	52 (85.2%)	22 (81.5%)		
Histological grade, n (%)			0.195	.659
I or II	40 (65.6%)	19 (70.4%)		
III	21 (34.4%)	8 (29.6%)		
ALN status (%)			4.154	.042
Positive	31 (50.8%)	20 (74.1%)		
Negative	30 (49.2%)	7 (25.9%)		
*HER2* positivity, n (%)			0.008	.930
* HER2* ^+^	22 (36.1%)	10 (37.0%)		
* HER2* ^−^	39 (63.9%)	17 (63.0%)		

Data are presented as mean ± SD, median (lower quartile, upper quartile), or number (%).

ALN = axillary lymph node, ER = estrogen receptor, *HER2* = human epidermal growth factor receptor 2, PR = progesterone receptor, SD = standard deviation.

**Table 2:** CBBCT findings of patients in the training and testing cohorts.

Finding	Training cohort (n = 61)	Testing cohort (n = 27)	*t*/*χ*^2^ value	*P* value
Breast density, n (%)			0.242	.623
a/b	26 (42.6%)	10 (37.0%)		
c/d	35 (57.4%)	17 (63.0%)		
No. of lesions, n (%)			0.871	.351
Single	39 (63.9%)	20 (74.1%)		
Multiple	22 (36.1%)	7 (25.9%)		
Tumor margins, n (%)			1.171	.279
Nonspiculated	43 (70.5%)	22 (81.5%)		
Spiculated	18 (29.5%)	5 (18.5%)		
Internal enhancement, n (%)			0.040	.980
Homogeneous	11 (18.0%)	5 (18.5%)		
Heterogeneous	44 (72.1%)	19 (70.4%)		
Rim enhancement	6 (9.8%)	3 (11.1%)		
CBBCT-associated non-mass enhancement, n (%)			1.057	.304
Present	12 (19.7%)	8 (29.6%)		
Absent	49 (80.3%)	19 (70.4%)		
Suspicious calcification, n (%)				
Present	22 (36.1%)	10 (37.0%)	0.008	.930
Absent	39 (63.9%)	17 (63.0%)		
Calcification morphology, n (%)				.373
Amorphous	16 (72.7%)	6 (60.0%)		
Coarse heterogeneous	6 (27.3%)	4 (40.0%)		
Vascular abnormalities, n (%)				
Present	46 (75.4%)	21 (77.8%)	0.058	.810
Absent	15 (24.6%)	6 (22.2%)		

### 3.2. Intra- and interobserver agreement for radiomics feature extraction

**Figure 3.**
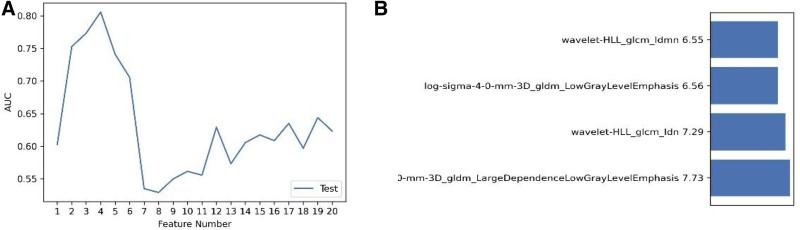
(A) Selected optimal features; (B) ultimately selected features.

The model based on these 4 features showed the highest AUC in the testing cohort (Fig. 3A). The selected features were as follows (Fig. 3B):

- log-sigma-4-0-mm-3D_gldm_LargeDependenceLowGrayLevelEmphasis
- log-sigma-4-0-mm-3D_gldm_LowGrayLevelEmphasis
- wavelet-HLL_glcm_Idmn
- wavelet-HLL_glcm_Idn

The intraobserver ICC of these 4 features ranged from 0.750 (95% confidence interval [CI], 0.531–0.870) to 0.840 (95% CI, 0.689–0.921), and the interobserver ICC ranged from 0.832 (95% CI, 0.641–0.921) to 0.997 (95% CI, 0.993–0.998). The results showed satisfactory repeatability and stability of feature extraction, both within and between the observers.

### 3.3. Prediction performance of machine learning models

**Figure 4.**
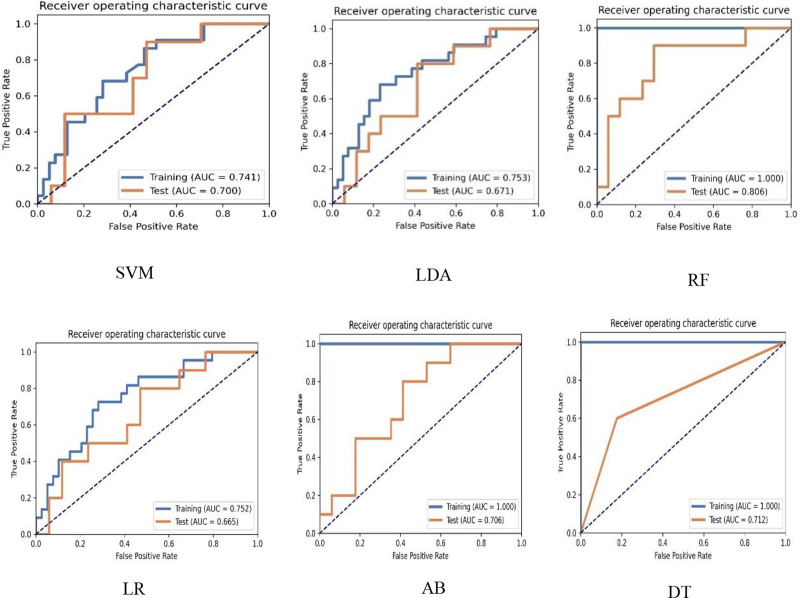
ROC curves of the SVM, LDA, RF, LR, AB, and DT classifiers in the training and testing cohorts. AB = adaboost, DT = decision tree, LDA = linear discriminant analysis, LR = logistic regression, RF = random forest, ROC = receiver operating characteristi curves, SVM = support vector machine.

The AUC values for SVM, linear discriminant analysis, RF, LR, AB, and DT were 0.741, 0.753, 1.000, 0.752, 1.000, and 1.000, respectively, in the training cohort and 0.700, 0.671, 0.806, 0.665, 0.706, and 0.712, respectively, in the testing cohort(Table 3). The DeLong test showed that the ROCs of RF, AB, and DT were significantly different from those of the training cohort (all *P* < .001) (Table 4). Notably, the RF model exhibited the highest predictive ability with an AUC of 0.806 in the testing cohort. For Sen and Spe, the RF model achieved values of 0.900 and 0.706, respectively. However, the DeLong test results for the testing cohort indicated no statistically significant differences in AUC among all models (Table 5). Figure 4 shows the ROC curves for the 6 ML models. When comparing the AUC of the RF model between the training and testing cohorts, the DeLong test showed a significant difference (*Z* = 2.105, *P* = .035).

**Table 3:** Prediction performance of machine learning models.

	Training cohort						Testing cohort					
ML algorithm	Acc	Sen	Spe	AUC	PPV	NPV	Acc	Sen	Spe	AUC	PPV	NPV
SVM	0.656	0.864	0.539	0.741	0.514	0.875	0.667	0.900	0.529	0.700	0.529	0.900
LDA	0.738	0.682	0.769	0.753	0.625	0.811	0.667	0.800	0.588	0.671	0.533	0.833
RF	1.000	1.000	1.000	1.000	1.000	1.000	0.778	0.900	0.706	0.806	0.643	0.923
LR	0.721	0.727	0.718	0.752	0.593	0.824	0.630	0.800	0.529	0.665	0.500	0.818
AB	1.000	1.000	1.000	1.000	1.000	1.000	0.667	0.800	0.588	0.706	0.533	0.833
DT	1.000	1.000	1.000	1.000	1.000	1.000	0.741	0.600	0.824	0.712	0.667	0.778

AB = AdaBoost, Acc = accuracy, AUC = area under the receiver operating characteristic curve, DT = decision tree, LDA = linear discriminant analysis, LR = logistic regression, ML = machine learning, NPV = negative predictive value, PPV = positive predictive value, RF = random forest, Sen = sensitivity, Spe = specificity, SVM = support vector machine.

**Table 4:** DeLong test results for each ROC curve in the training cohort.

Comparison	*Z* value	*P* value
SVM versus LDA	0.539	.590
SVM versus RF	4.030	<.001
SVM versus LR	0.363	.717
SVM versus AB	4.030	<.001
SVM versus DT	4.030	<.001
LDA versus RF	3.840	<.001
LDA versus LR	0.271	.787
LDA versus AB	3.840	<.001
LDA versus DT	3.840	<.001
RF versus LR	3.897	<.001
RF versus AB	0.000	1.000
RF versus DT	0.000	1.000
LR versus AB	3.897	<.001
LR versus DT	3.897	<.001
AB versus DT	0.000	1.000

AB = AdaBoost, Acc = accuracy, AUC = area under the receiver operating characteristic curve, DT = decision tree, LDA = linear discriminant analysis, LR = logistic regression, RF = random forest, ROC = receiver operating characteristi curves, Sen = sensitivity, Spe = specificity, SVM = support vector machine.

**Table 5:** DeLong test results for each ROC curve for the test cohorts.

Comparison	*Z value*	*P value*
SVM versus LDA	1.130	.258
SVM versus RF	−1.088	.277
SVM versus LR	1.275	.202
SVM versus AB	−0.086	.932
SVM versus DT	−0.097	.923
LDA versus RF	−1.304	.192
LDA versus LR	0.227	.820
LDA versus AB	−0.536	.592
LDA versus DT	−0.345	.730
RF versus LR	1.289	.198
RF versus AB	0.995	.320
RF versus DT	1.096	.273
LR versus AB	−0.608	.543
LR versus DT	−0.376	.707
AB versus DT	−0.055	.956

AB = AdaBoost, Acc = accuracy, AUC = area under the receiver operating characteristic curve, DT = decision tree, LDA = linear discriminant analysis, LR = logistic regression, RF = random forest, ROC = receiver operating characteristi curves, Sen = sensitivity, Spe = specificity, SVM = support vector machine.

## 4. Discussion

This retrospective study aimed to assess the potential of certain radiomic features derived from CE-CBBCT in predicting *HER2*-positive IBC. After careful analysis, we identified 4 optimal features that demonstrated satisfactory reproducibility and reliability. The RF method showed high efficiency in this study. These results provide valuable insights for further research and improving the diagnosis and treatment of patients with IBC.

To our knowledge, this is the first study to explore the association between CE-CBBCT image-derived radiomic features and *HER2*-positive IBC. Previous studies have explored the use of radiomics to predict *HER2*-positive BC, with varying AUC levels. In a study conducted by Ma et al,^[15]^ mammographic radiomics signatures with the potential to determine *HER*2 positivity were identified with an AUC of 0.784. Similarly, Ferrea et al^[16]^ reported an excellent predictive performance using ultrasound images, with an AUC of 0.778. Another study by Fang et al^[18]^ demonstrated that combining diffusion-weighted imaging and dynamic contrast-enhanced MRI significantly improved diagnostic performance in identifying HER2 positivity, with an AUC of 0.79. Our findings were slightly higher than those of the previous studies. Disparities between 2D mammography, ultrasound images, and 3D CE-CBBCT images could contribute to these inconsistencies. CE-CBBCT can capture intricate details that digital mammography and ultrasound cannot, including the tumor’s precise location, size, and shape, the invasion of adjacent tissue, intratumoral hemorrhage, necrosis, and other complex features. In addition, CE-CBBCT provides information regarding calcifications that are absent in MRI.

The 4 features with the highest AUC were finally selected, encompassing 2 features from the wavelet transformation and 2 from the LoG. Wavelet decomposition was used to calculate the intensity and texture attributes of the original image, thereby obtaining wavelet transform features. These features are mainly concentrated in different frequency bands of tumor volume.^[32]^ Images transformed through wavelet processing demonstrated the uniformity and coherence of tumor texture characteristics. This is beneficial for effectively exploring heterogeneity within the tumor and subtle variations in grayscale and texture levels.^[33]^ Moreover, higher-order statistical features obtained from wavelet transformation have the potential to provide valuable insights into quantifying the biological heterogeneity of tumors from multidimensional perspectives.^[28,34]^ However, a precise interpretation of the relationship between these complex parameters and the biological functions of tumors requires further investigation. Li et al,^[35]^ who conducted research on extracting wavelet features to predict *HER2* positivity, proposed that wavelet features contain more detailed BC information. These features have been identified as crucial components of radiomics models. Log^[36]^ is a measure of the second-order spatial derivative of an image and is used to emphasize areas with rapid changes in the display intensity. All of the features above are considered higher-order features that can more convincingly elucidate disparities in the tumor space. Previous studies^[25,37]^ found that extracting higher-order features facilitates the identification of different molecular subtypes of breast lesions.

ML algorithms are crucial for identifying radiomic features associated with outcome variables. The most effective predictive models can be extracted by formulating predictive models using various ML algorithms and comparing their performance. In previous studies, several scholars^[11,17,38,39]^ compared different ML algorithms to identify the most effective model for predicting the outcome variables. These studies consistently indicated that RF models outperformed other ML algorithms. For instance, Sheng et al^[17]^ conducted a study predicted HER2 positivity in patients with BC using MRI radiomic features. They evaluated 5 ML models and found that the RF model exhibited a superior performance. In the testing cohort, the RF model achieved an AUC of 0.8054, surpassing the AUCs of other models: 0.7029 (LR), 0.7164 (naïve Bayes), 0.7617 (SVM), and 0.7459 (extreme Gradient Boosting). Similarly, Ma et al^[39]^ discovered that the RF classifier was significantly better at predicting HER2 positivity than other classifiers. The RF classifier yielded an AUC of 0.855, whereas the AUCs of the other classifiers were 0.818 (naïve Bayes), 0.835 (LR), 0.838 (DT), and 0.834 (k-nearest neighbor). In our study, the RF model demonstrated superior performance compared to the other 5 models in the testing cohort, achieving an AUC of 0.806, which indicated high classification efficiency. The sensitivity of the model was 0.900, suggesting that it was effective in detecting positive samples.

However, this study has some limitations. First, it is crucial to emphasize that this study was retrospective and relied on a limited cohort of patients recruited from a single institution. This might have introduced selection bias, affecting the generalizability of our findings. To address this issue, future prospective studies should be conducted, incorporating more samples from multiple centers, to validate and bolster the outcomes of our research. Moreover, we achieved ROI segmentation through manual delineation, which is time consuming. To overcome this limitation, it is imperative to develop and implement semiautomatic segmentation methods in future studies to enhance the efficiency of the segmentation process and to increase its reproducibility. Additionally, our models were constructed solely based on radiomic features derived from CE-CBBCT scans. However, the potential benefits of using an integrated model that incorporates multimodal medical images are worth investigating. This approach can potentially enhance the predictive efficiency of our model in future studies. Notably, our investigation depended solely on classical supervised classification algorithms to explore the predefined radiomic characteristics. To gain a more comprehensive understanding, further research should investigate using deep learning algorithms to extract more informative and abstract features from CE-CBBCT images. Examining these features could provide valuable insights and advance the field of radiomic analysis.

## 5. Conclusions

ML models based on CE-CBBCT radiomic features have the potential to predict *HER2*^+^ IBC. Among these, the RF model demonstrated the best diagnostic performance.

## Acknowledgments

We thank LetPub (www.letpub.com) for the linguistic assistance in preparing this manuscript.

## Author contributions

**Conceptualization:** Xianfei Chen.

**Data curation:** Xianfei Chen.

**Formal analysis:** Xianfei Chen, Danke Su.

**Funding acquisition:** Xianfei Chen.

**Investigation:** Xianfei Chen.

**Methodology:** Xianfei Chen, Minghao Li.

**Project administration:** Danke Su.

**Resources:** Danke Su.

**Software:** Minghao Li, Xueli Liang.

**Supervision:** Danke Su.

**Validation:** Xianfei Chen, Xueli Liang.

**Visualization:** Xianfei Chen, Minghao Li.

**Writing – original draft:** Xianfei Chen.

**Writing – review & editing:** Danke Su.

## Supplementary Material


