# Supplementary material for: Machine learning model based on the radiomics features of CE-CBBCT shows promising predictive ability for HER2-positive BC
Source: Medicine (Baltimore). 2025 Sep 12;104(37):e44300. doi: 10.1097/MD.0000000000044300 (PMC12440477; doi:10.1097/MD.0000000000044300)
Supplement: Supplementary file 1 [file medi-104-e44300-s001.docx]

**The parameters for “image preprocessing steps”**

setting:

# Normalization:

normalize: true

normalizeScale:100 # This allows you to use more or less the same bin width.

# Resampling:

# If slices are very thin (~1mm), such as in 3D scanned (isotropic) volumes, resampled pixelspacing may be reduced to

#(1,1,1). Furthermore, in case of isotropic volumes, consider disabling resampling.

# On a side note: increasing the resampled spacing forces PyRadiomics to look at more coarse textures, which may or

# may not increase the accuracy and stability of your extracted features.

interpolator:'sitkBSpline'

#resampledPixelSpacing:[2，2，2]

resampledPixelspacing:[1,1,1]

# Mask validation:

# correctMask and geometryTolerance are not needed, as both image and mask are resampled, if you expect very small

# masks, consider to enable a size constraint by uncommenting settings below:

#minimumROIDimensions:2

#minimumRoIsize:50

# Image discretization:

# The ideal number of bins is somewhere in the order of 16-128 bins. A possible way to define a good binwidt is to

# extract firstorder:Range from the dataset to analyze, and choose a binwidth so, that range/binwidth remains approximately

# in this range of bins.

binwidth: 5
